# A remote Tai Chi program for diverse older adults with multisite pain during the COVID-19 pandemic

**DOI:** 10.1093/geroni/igab046.3553

**Published:** 2021-12-17

**Authors:** Yael Koren, Suzanne Leveille, Catarina Ambrizzi Moraes, William Butts, Peter Wayne, Gloria Yeh, Tongjian You

**Affiliations:** 1 University of Massachusetts Boston, University of Massachusetts Boston, Massachusetts, United States; 2 Harvard Medical School, Chestnut Hill, Massachusetts, United States; 3 Harvard Medical School, Boston, Massachusetts, United States

## Abstract

The goal of this study was to evaluate feasibility and acceptability of a remote Tai Chi program in diverse older adults with multisite pain and risk for falls during the COVID-19 pandemic. Adults aged ≥65y living in diverse Boston neighborhoods were invited through mailed letters to participate in a recruitment and screening survey. Eligible adults were re-contacted to join a 4-week Tai Chi or light exercise program offered online twice weekly. We conducted pre- and post-interviews to assess pain characteristics, fall risk, computer use, and satisfaction with the program. Primary outcomes were class attendance, experience, and program safety. Among 335 survey respondents, 105 (31%) were eligible based on multisite pain and fall history or cane/walker use. Of the eligible respondents, average age was 74y, 75% were women, 62% were Black, and 31% had high school education or less. We assigned 32 participants to 4 Tai Chi (Yang-style Tai Chi tailored to older adults with pain) or 2 light exercise (stretching and strength exercise) groups conducted via zoom; of these, 24 (75%) completed the program. Overall, 79% attended ≥6 of 8 classes. There were no adverse events reported. Regarding experiences with remote exercise, 67% reported it was very easy to join, 88%, very easy to see the instructor and 83%, very easy to participate. For future planning, 29% prefer remote classes, 33% prefer in-person classes, and 38% could do either. In conclusion, remote exercise programming is safe and feasible for diverse older adults who have multisite pain and risk of falls.

